# Supplementary material for: The Effect of Glycidyl Azide Polymer Grafted Tetrafunctional Isocyanate on Polytriazole Polyethylene Oxide-Tetrahydrofuran Elastomer and its Propellant Properties
Source: Polymers (Basel). 2020 Jan 31;12(2):278. doi: 10.3390/polym12020278 (PMC7077387; doi:10.3390/polym12020278)
Supplement: Supplementary file 1 [file polymers-12-00278-s001.zip › polymers-671921-supplementary.pdf]

Supporting information

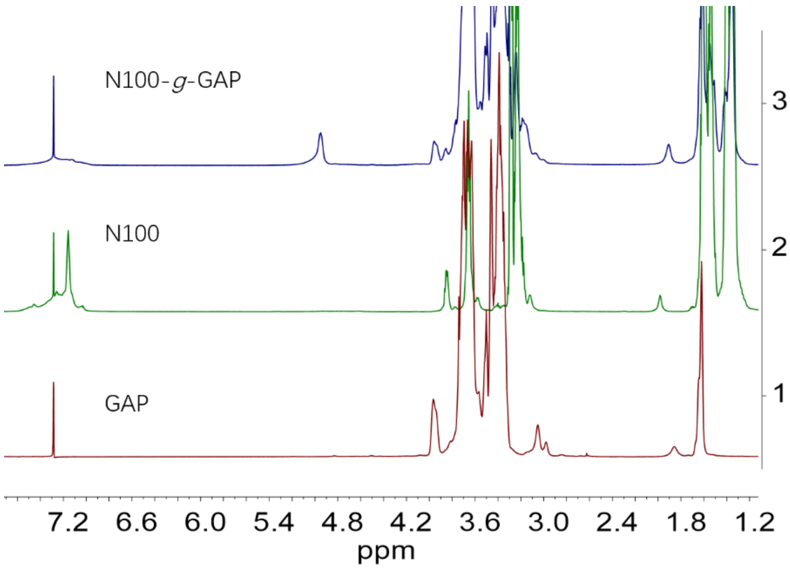

Figure 1.^{1}H NMR analysis of N100-g-GAP, N100, and GAP.

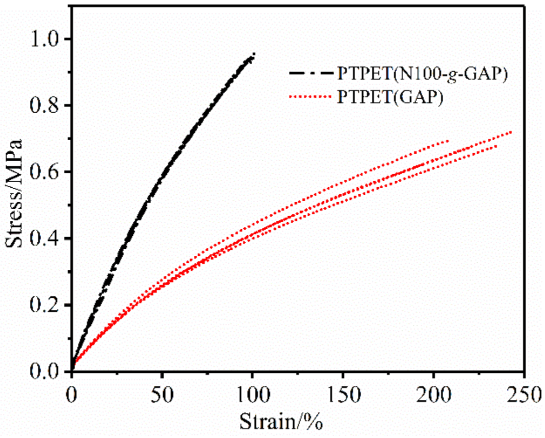

Figure 2. Stress–strain curves of the PTPET elastomer prepared by N100-*g*-GAP and GAP (*Mn* = 4,000 g/mol) at 20 ℃.

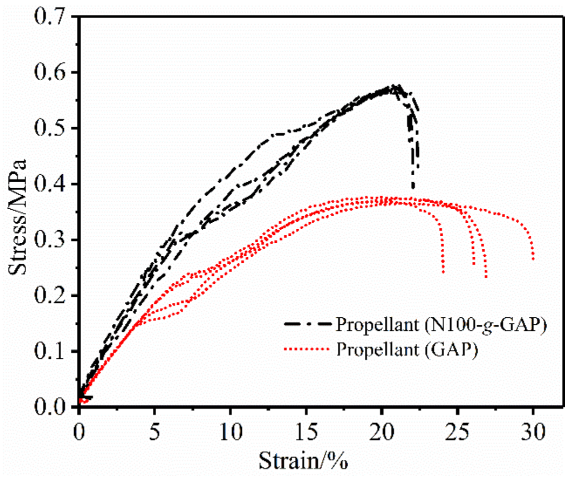

Figure 3. Stress–strain curves of the PTPET propellants prepared by N100-*g*-GAP and GAP (*Mn* = 4,000 g/mol) at 20 ℃.
